# Time-Frequency Analysis of Chemosensory Event-Related Potentials to Characterize the Cortical Representation of Odors in Humans

**DOI:** 10.1371/journal.pone.0033221

**Published:** 2012-03-09

**Authors:** Caroline Huart, Valéry Legrain, Thomas Hummel, Philippe Rombaux, André Mouraux

**Affiliations:** 1 Institute of Neuroscience (IONS), Université Catholique de Louvain, Brussels, Belgium; 2 Department of Otorhinolaryngology, Cliniques Universitaires Saint-Luc, Brussels, Belgium; 3 Department of Experimental Clinical and Health Psychology, Ghent University, Ghent, Belgium; 4 Department of Otorhinolaryngology, Dresden Medical School, Technical University, Dresden, Germany; Alexander Flemming Biomedical Sciences Research Center, Greece

## Abstract

**Background:**

The recording of olfactory and trigeminal chemosensory event-related potentials (ERPs) has been proposed as an objective and non-invasive technique to study the cortical processing of odors in humans. Until now, the responses have been characterized mainly using across-trial averaging in the time domain. Unfortunately, chemosensory ERPs, in particular, olfactory ERPs, exhibit a relatively low signal-to-noise ratio. Hence, although the technique is increasingly used in basic research as well as in clinical practice to evaluate people suffering from olfactory disorders, its current clinical relevance remains very limited. Here, we used a time-frequency analysis based on the wavelet transform to reveal EEG responses that are not strictly phase-locked to onset of the chemosensory stimulus. We hypothesized that this approach would significantly enhance the signal-to-noise ratio of the EEG responses to chemosensory stimulation because, as compared to conventional time-domain averaging, (1) it is less sensitive to temporal jitter and (2) it can reveal non phase-locked EEG responses such as event-related synchronization and desynchronization.

**Methodology/Principal Findings:**

EEG responses to selective trigeminal and olfactory stimulation were recorded in 11 normosmic subjects. A Morlet wavelet was used to characterize the elicited responses in the time-frequency domain. We found that this approach markedly improved the signal-to-noise ratio of the obtained EEG responses, in particular, following olfactory stimulation. Furthermore, the approach allowed characterizing non phase-locked components that could not be identified using conventional time-domain averaging.

**Conclusion/Significance:**

By providing a more robust and complete view of how odors are represented in the human brain, our approach could constitute the basis for a robust tool to study olfaction, both for basic research and clinicians.

## Introduction

In 1978, Kobal and Plattig [1] introduced a device capable of delivering transient chemosensory stimuli over the olfactory neuroepithelium of the nasal mucosa, without concurrent mechanical and/or thermal stimulation. The stimulator opened new perspectives for exploring how the human brain processes odors, through the non-invasive recording of time-locked chemosensory event-related brain potentials (ERPs) [2], [3], [4], [5]. Until then, the use of electrophysiological techniques for the functional exploration of olfaction in humans had remained limited, mainly because of the lack of adequate methods to produce a selective, controlled and transient chemosensory stimulus [6], and the fact that pioneering studies had yielded conflicting results [7], [8], [9]. Early stimulation methods relied on the delivery of brief air puffs containing a given odorant. Inevitably, the sudden increase in airflow associated with the presentation of the air puff activated mechanically-sensitive trigeminal afferents, and determining whether the elicited brain responses were triggered by the odorant or by concurrent mechanical stimulation of the nasal mucosa was difficult. In contrast, the device reported by Kobal and Plattig [1] delivers pulses of odorant embedded within a constant airflow, thus avoiding any concomitant mechanical stimulation of the nasal mucosa, making it possible to study the brain responses related specifically to the activation of chemosensitive afferents. Furthermore, using specific odorants, the device can be used to activate olfactory and trigeminal chemosensory receptors relatively selectively. For example, 2-phenylethanol can be used to elicit chemosensory ERPs related to activation of olfactory afferents, while gaseous CO_2_ can be used to elicit chemosensory ERPs related to the activation of trigeminal afferents [10], [11], [12].

The ability to characterize, non-invasively, the cortical processing of chemosensory stimuli in humans is not only of interest for research in the neuroscience of odor perception. Indeed, the technique is used as a clinical diagnostic tool [13], [14]. Recent studies have shown that disorders of olfaction are extremely common (affecting up to 20% of the general population; [15]) and, although olfactory dysfunction is often unnoticed, it has been shown to impact significantly on the quality of life [16]. Finally, several studies have underlined the potential diagnostic value of olfactory dysfunction, which could constitute a premonitory sign of several neurodegenerative diseases, in particular, idiopathic Parkinson's disease and Alzheimer-type dementia [17], [18], [19], [20], [21], [22].

Until now, the electroencephalographic (EEG) responses to chemosensory stimulation have been identified mainly using across-trial averaging in the time domain, a procedure which cancels out changes in the EEG signal that are not time-locked to the stimulus onset and that are not strictly stationary across trials and, thereby, enhances the signal-to-noise ratio of time-locked ERPs [11], [23], [24], [25], [26]. Using such an approach, the EEG responses to chemosensory stimulation have been characterized as a negative wave peaking approximately 320–500 ms after stimulus onset (N1), followed by a positive wave peaking approximately 450–800 ms after stimulus onset (P2 and/or P3) [2], [11], [27], [28], [29]. Regardless of the type of chemosensory stimulus, all of these responses are maximally recorded over the scalp midline, whereby the N1-P2 complex elicited by olfactory stimulation is sometimes described as displaying a slightly more parietal scalp distribution than the N1-P2 complex elicited by trigeminal stimulation [2], [30], [31].

Although it has been demonstrated that the latency and, to a lesser extent, the amplitude of chemosensory ERPs are relatively reproducible within subjects [32]; chemosensory ERPs - in particular, olfactory ERPs – usually exhibit a very low signal-to-noise ratio [10], [26], [33]. For example, Lötsch and Hummel [10] could not identify any reproducible olfactory ERP in approximately 30% of normosmic subjects. Hence, although the recording of chemosensory ERPs is considered as a technique having great potential, its current usefulness remains very limited, particularly in the context of clinical diagnosis.

Here, we hypothesized that the low signal-to-noise ratio of chemosensory ERPs could at least in part be due to an important amount of temporal jitter affecting the brain responses to chemosensory stimulation. Indeed, the existence of a significant amount of temporal jitter would imply that the elicited EEG responses are no longer strictly stationary across trials and, hence, that these EEG responses will be distorted or even cancelled-out using conventional across-trial averaging procedures performed in the time domain. The hypothesized jitter would result from the different steps separating the onset of the chemosensory stimulus and the generation of cortical responses. Using patch clamp recordings, the currents elicited by brief pulses of odors have been characterized by latencies ranging from 175 to 600 ms [34], thus indicating that the intracellular chemosensory transduction steps can constitute a significant source of latency jitter. Furthemore, Guetchell et al. [35] showed a relationship between odor concentration and response latency in preparations including a mucus layer, most probably because diffusion speed across the mucus layer is dependent on odor concentration. Hence, inevitable trial-by-trial variations in the physical properties of the olfactory stimulus – in particular, the relative position of the tube ending used to deliver the stimulus relative to the olfactory epithelium – may be expected to induce significant trial-by-trial latency jitter in the neural activity triggered within the olfactory receptor neurons. An additional significant source of variability may be the influence of the respiratory dynamics on bulbar and cortical activity (reviewed in [36]).

For this reason, in order to increase the signal-to-noise ratio of chemosensory EEG responses, we used an alternative approach to reveal EEG activity that is induced by the stimulus (and, thereby, related to its cortical processing), but not sufficiently stationary across trials to be revealed by classic averaging in the time domain. The approach relied on a time-frequency decomposition of single-trial EEG epochs performed using the continuous wavelet transform [25], [37], such as to express signal amplitude as a function of time and frequency, regardless of phase [25], [37]. Across-trial averaging of the obtained time-frequency maps of EEG amplitude yielded the average signal amplitude as a function of latency and frequency, within which transient increases or decreases of EEG amplitude could be identified. As suggested by the results of previous studies [37], [38], the approach may be expected to reveal ERPs even if these are not strictly stationary across trials because of a significant amount of latency jitter. Hence, the approach could be more effective at revealing chemosensory ERPs. Furthermore, because across-trial averaging in the time-frequency domain enhances time-locked EEG changes regardless of whether these changes are phase-locked across trials, the procedure can effectively enhance stimulus-induced modulations of the amplitude of ongoing EEG oscillations (event-related synchronization and desynchronization, ERS and ERD respectively), hypothesized to reflect mechanisms involved in cortical activation and deactivation underlying sensory-motor and cognitive functions [39], [40], [41].

Using this approach, the aims of the present study were thus (1) to characterize for the first time the phase-locked and non phase-locked EEG responses to trigeminal and olfactory stimulation and (2) to examine whether this approach may be used to enhance the signal-to-noise ratio of the obtained EEG responses and, thereby, increase their potential usefulness as a research and clinical diagnostic tool to study olfaction in humans.

## Results

### Across-trial averaging in the time domain

**Figure 1:**
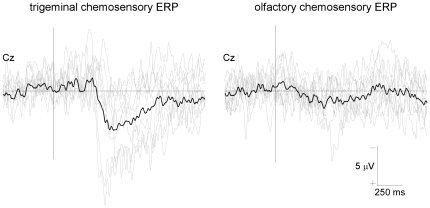
Trigeminal and olfactory chemosensory ERPs. Trigeminal and olfactory chemosensory ERPs recorded at the scalp vertex (Cz vs. A1A2) in 11 healthy normosmic volunteers. Gaseous CO_2_ (50% v/v) was used to selectively activate trigeminal afferents. 2-Phenylethanol (50%v/v) was used to selectively activate olfactory afferents. 60 stimuli were presented, lasting 200 ms (20-ms rise-time), separated by a 30 s inter-stimulus interval. Individual ERP waveforms are shown in light grey, while the group-level average waveform is shown in black. Note that trigeminal chemosensory stimulation elicited a clear negative-positive complex (TRI-N1/TRI-P2), contrasting with the low signal-to-noise ratio of the response elicited by olfactory chemosensory stimulation, which was clearly identifiable in only a few subjects.

**Figure 2:**
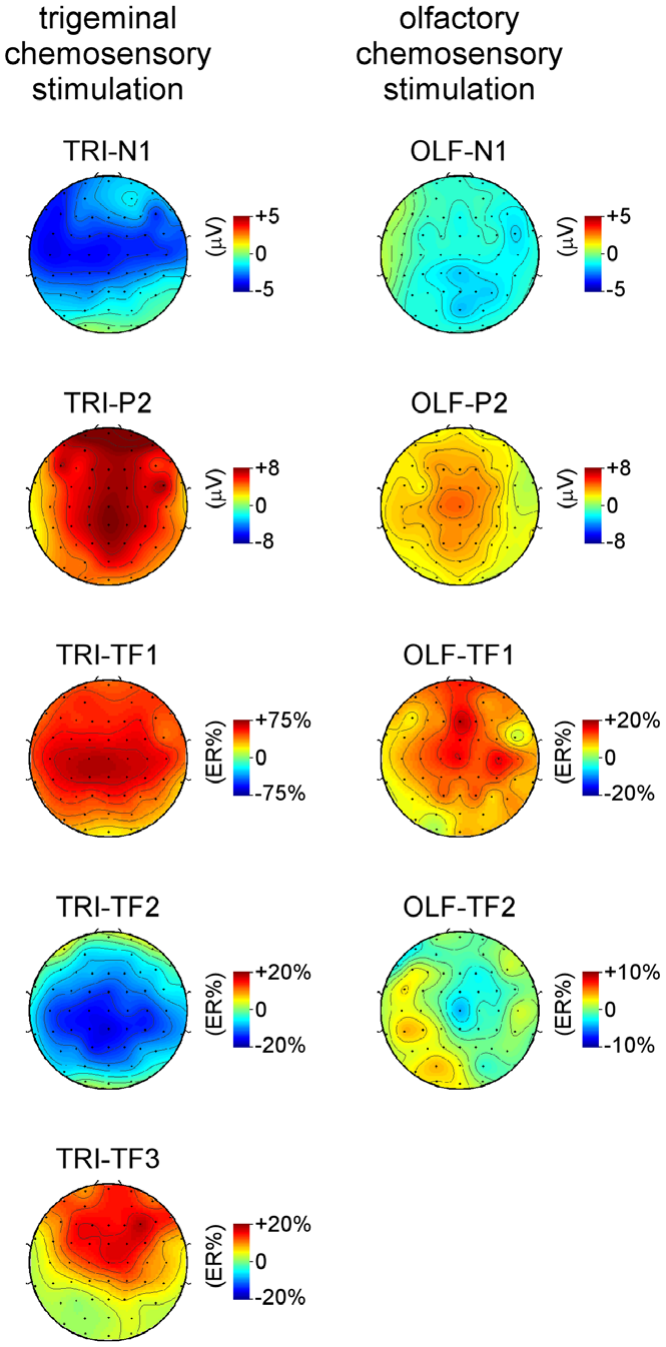
Scalp distribution of the EEG responses elicited by trigeminal and olfactory chemosensory stimulation. The scalp distribution of the EEG responses identified using across-trial averaging in the time domain (TRI-N1, TRI-P2, OLF-N1, OLF-P2) are expressed in microvolts, whereas the scalp distribution of the EEG responses identified using across-trial averaging in the time-frequency domain (TRI-TF1, TRI-TF2, TRI-TF3, OLF-TF1, OLF-TF2) are expressed as relative percentage increase or decrease relative to baseline.

*Trigeminal stimulation*. As shown in the group-level average waveforms displayed in Figure 1, trigeminal stimulation elicited a negative deflection (TRI-N1: 391±55 ms) followed by a positive deflection (TRI-P2: 554±57 ms). The scalp distribution of both peaks was widely distributed over the two hemispheres, and maximal at the scalp vertex (Figure 2). At electrode Cz, TRI-N1 amplitude was 3.5±2.0 µV and TRI-P2 amplitude was 7.0±4.5 µV.

**Figure 3:**
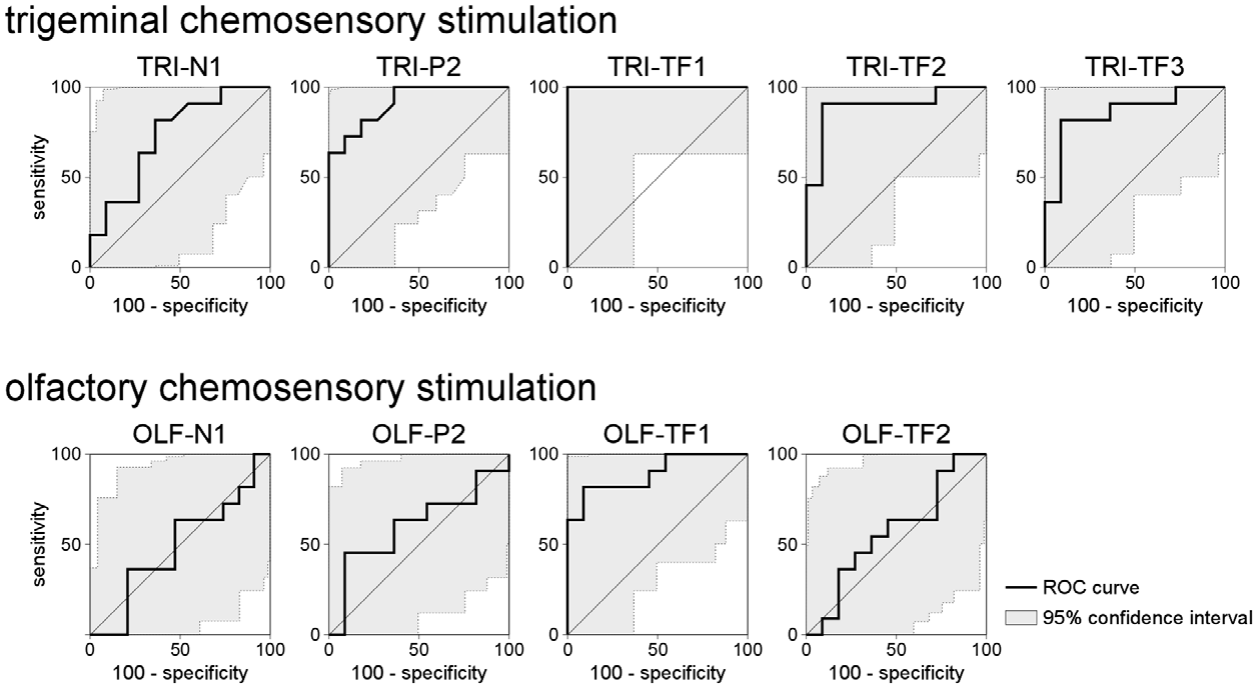
Receiver Operating Characteristic (ROC) curves. ROC curves were computed to estimate the discrimination performance (ability to discriminate between presence vs. absence of stimulation) of each of the different EEG responses identified using across-trial averaging in the time domain (TRI-N1, TRI-P2, OLF-N1, OLF-P2) and across-trial averaging in the time-frequency domain (TRI-TF1, TRI-TF2, TRI-TF3, OLF-TF1, OLF-TF2). The shaded areas represent the 95% confidence interval of the obtained curves.

The measure of TRI-N1 peak amplitude had a marginal discrimination performance, with an AUC of 0.73±0.11 (p = 0.038) (Table 1, Figure 3). This measure was thus only marginally efficient for discriminating between presence vs. absence of a trigeminal ERP and, hence, its relevance can be questioned.

**Table 1:** Discrimination performance of the phase-locked and non phase-locked EEG responses to trigeminal and olfactory chemosensory stimulation.

ROI	Latency (ms)	Frequency (Hz)	AUC	p-value	Sensitivity (%)	Specificity (%)
TRI-N1	391±55		0.73±0.11	0.038	81.2	63.6
TRI-P2	554±57		0.91±0.06	<0.0001	81.2	81.2
TRI-TF1	490±74	3.5±0.9	1.00±0.00	<0.0001	100.0	100.0
TRI-TF2	811±140	10.0±1.2	0.89±0.08	<0.0001	90.9	90.9
TRI-TF3	383±50	12.8±2.8	0.86±0.09	<0.0001	81.8	90.8
OLF-N1	397±27		0.50±0.13	0.975	36.4	31.8
OLF-P2	616±109		0.60±0.13	0.429	45.4	90.9
OLF-TF1	729±241	5.1±1.4	0.89±0.07	<0.0001	81.8	90.9
OLF-TF2	1190±98	9.6±1.7	0.57±0.13	0.589	45.4	72.7

In contrast, the measure of TRI-P2 peak amplitude had a high discrimination performance, with an AUC of 0.91±0.06 (p<0.0001). Using the cutoff value associated with the greatest Youden index, the measure of TRI-P2 peak amplitude had a sensitivity of 81.2% and a specificity of 81.2% for discriminating between presence vs. absence of a trigeminal ERP (Table 1, Figure 3).

*Olfactory stimulation*. As shown in the group-level average waveforms displayed in Figure 1, olfactory stimulation elicited clearly identifiable negative and positive peaks in only a few subjects. In these subjects, the olfactory ERP appeared maximal at the vertex with a slightly more posterior distribution for OLF-N1 as compared to OLF-P2 (Figure 2).

At electrode Cz, the estimated OLF-N1 latency and amplitude was 397±27 ms and −0.9±1.3 µV, whereas the estimated OLF-P2 latency and amplitude was 616±109 ms and 4.2±2.4 µV. Neither the measure of OLF-N1 amplitude nor the measure of OLF-P2 amplitude were able to discriminate significantly between presence vs. absence of an olfactory response (OLF-N1 amplitude: AUC = 0.50±0.13, p = 0.975; OLF-P2 amplitude: AUC = 0.60±0.13, p = 0.429) (Table 1, Figure 3).

### Across-trial averaging in the time-frequency domain

#### Trigeminal stimulation

**Figure 4:**
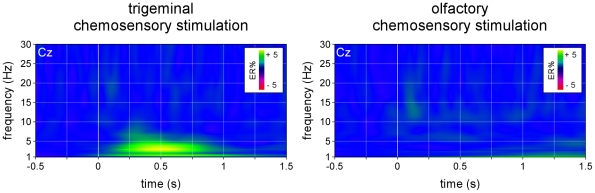
Time-frequency representation of the phase-locked EEG responses to trigeminal and olfactory chemosensory stimulation (CWT-AVERAGE). The time-frequency transform of the waveforms obtained by performing conventional across-trial averaging in the time domain was used to identify EEG responses that were phase-locked across trials, as these are preserved by the averaging procedure. Signal amplitude (group-level average, electrode Cz vs. A1A2) is expressed as percentage increase or decrease relative to baseline (−0.4 to −0.1 s) (ER%). Note that the trigeminal chemosensory ERP is mainly represented by an increase of low-frequency activities (1–5 Hz). Also note the lack of a clear EEG response following olfactory stimulation.

**Figure 5:**
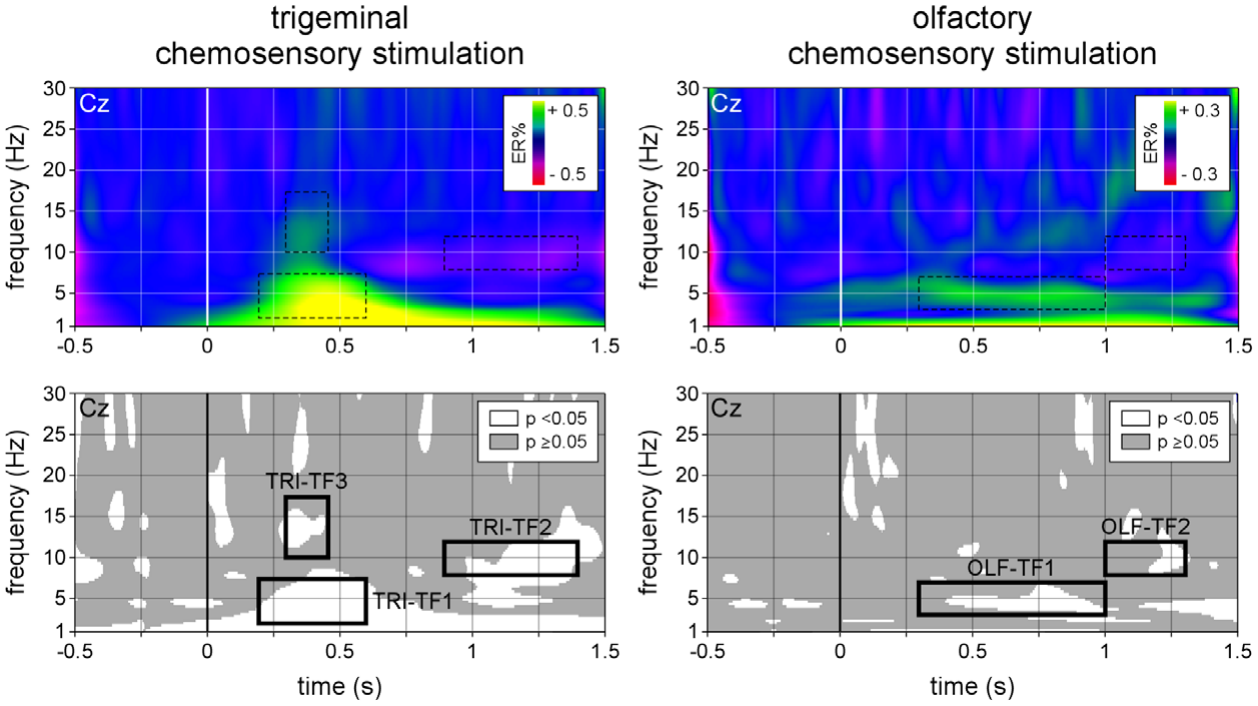
Time-frequency representation of the non-phase locked EEG responses to trigeminal and olfactory chemosensory stimulation (CWT-SINGLE). Non-phase locked EEG responses were identified by performing across-trial averaging in the time-frequency domain, a procedure which enhances time-locked EEG responses regardless of whether they are phase-locked to the onset of the stimulus. The upper panels show the group-level average time-frequency maps of oscillation amplitude (group-level average; electrode Cz vs. A1A2), expressed as percentage increase or decrease relative to baseline (−0.4 to −0.1 s) (ER%). Note that, in addition to the phase-locked EEG response (TRI-TF1), trigeminal stimulation also elicits event-related desynchronization in the alpha-band (TRI-TF2), as well as a transient and early increase of signal amplitude centered around 10–15 Hz (TRI-TF3). Also note that olfactory stimulation elicits a long-lasting non phase-locked increase of signal amplitude centered around 5 Hz (OLF-TF1), possibly followed by event-related desynchronization in the alpha-band (OLF-TF2). The lower panels highlight areas of the time-frequency matrix where signal amplitude deviated significantly from baseline (one-sample t-test).

As shown in the CWT-AVERAGE transform, the time-frequency representation of the phase-locked trigeminal chemosensory ERP consisted mainly in an increase of signal amplitude peaking approximately 400 ms after stimulus onset, and centered at relatively low frequencies (approximately 1–5 Hz) (Figure 4). In addition to (1) this low-frequency phase-locked EEG response, the CWT-SINGLE transform revealed that trigeminal stimulation also elicited (2) a significant long-lasting desynchronization of alpha-band (8–12 Hz) oscillations starting approximately 600 ms after stimulus onset and (3) a significant non phase-locked increase in EEG signal amplitude peaking approximately 350 ms after stimulus onset and centered around 10–15 Hz (Figure 5).

Based on these observations, three distinct time-frequency ROIs were defined as follows. TRI-TF1: 200–600 ms and 2–7.5 Hz, circumscribing the time-frequency representation of the phase-locked trigeminal ERP. TRI-TF2: 900–1400 ms and 8–12 Hz, circumscribing the stimulus-induced desynchronization of alpha-band EEG oscillations. TRI-TF3: 300–450 ms and 10–17.5 Hz, circumscribing the non phase-locked increase in EEG power centered around 10–15 Hz (Figure 5).

The scalp topography of TRI-TF1 was maximal at the vertex (Figure 2). At electrode Cz, the activity within TRI-TF1 peaked at 490±74 ms and 3.5±0.9 Hz. The measurement of TRI-TF1 amplitude at electrode Cz and Pz had a perfect discrimination performance (AUC = 1.00±0.00, p<0.0001) (Table 1, Figure 3). TRI-TF1 amplitude was thus able to discriminate between presence vs. absence of a response with a sensitivity and specificity of 100%. As compared to the measures of peak amplitude performed in the time domain, the discrimination performance of TRI-TF1 amplitude was significantly greater than the discrimination performance of TRI-N1 amplitude (difference in AUC: 0.27±0.11, p = 0.016), but was not significantly different from the discrimination performance of TRI-P2 amplitude (difference in AUC: 0.09±0.136, p = 0.133) (Table 1).

As compared to the scalp topographies of TRI-TF1, the scalp topography of TRI-TF2 extended more towards posterior regions (Figure 2). At electrode Pz, maximum desynchronization was observed at 811±140 ms and 10.0±1.2 Hz. The discrimination performance of TRI-TF2 was significant (AUC = 0.89±0.08, p<0.0001) (Table 1, Figure 3). Using the cutoff value associated with the greatest Youden index, the measure of TRI-TF2 amplitude had a sensitivity of 90.9% and a specificity of 90.9% for discriminating between presence vs. absence of an EEG response to trigeminal stimulation. This discrimination performance was not significantly different from the discrimination performance of TRI-N1 amplitude (difference in AUC = 0.16±0.15, p = 0.277) and TRI-P2 amplitude (difference in AUC = 0.02±0.64, p = 0.746) (Table 1).

Such as the scalp topography of TRI-TF1, the scalp topography of TRI-TF3 was maximal over the vertex (Figure 2). At electrode Cz, the response peaked at 383±50 ms and 12.8±2.8 Hz. This peak latency was significantly different from the TRI-TF1 peak latency (t_10_ = 5.04; p = 0.0005) and from TRI-P2 peak latency (t_10_ = 2.83; p = 0.018), but was not significantly different from TRI-N1 peak latency (t_10_ = 0.50; p = 0.629). The discrimination performance of TRI-TF3 amplitude measured at Cz was significant (AUC = 0.86±0.09, p<0.0001) (Table 1, Figure 3). Using the cutoff value associated with the greatest Youden index, the measure of TRI-TF3 amplitude had a sensitivity of 81.8% and a specificity of 90.9% for discriminating between presence vs. absence of a trigeminal response. As compared to the measures of peak amplitude performed in the time domain, the discrimination performance of TRI-TF3 amplitude was not significantly different from the discrimination performance of TRI-N1 amplitude (difference in AUC = 0.13±0.14, p = 0.343) and TRI-P2 amplitude (difference in AUC = 0.05±0.11, p = 0.633) (Table 1).

#### Olfactory stimulation

As shown in the CWT-AVERAGE transform, olfactory chemosensory stimulation did not elicit any clearly circumscribed phase-locked increase in EEG signal power (Figure 4). In contrast, the CWT-SINGLE transform revealed that olfactory stimulation did elicit a number of significant non phase-locked increases and decreases in EEG power, consisting mainly in (1) a long-lasting increase in the amplitude of low frequencies extending from approximately 300 ms to 1000 ms after stimulus onset, centered around 5 Hz, followed by (2) a desynchronization of alpha-band EEG oscillations, starting approximately 1000 ms after stimulus onset (Figure 5).

Based on these observations, two distinct time-frequency ROIs were defined as follows. OLF-TF1: 300–1000 ms and 3–7 Hz, circumscribing the long-lasting increase in low frequency amplitude extending centered around 5 Hz. OLF-TF2 1000–1300 ms and 8–12 Hz, circumscribing the stimulus-induced desynchronization of alpha-band EEG oscillations (Figure 5).

The scalp topography of OLF-TF1 was maximal over fronto-central regions (Figure 2). At electrode Fz, the response peaked at 729±242 ms and 5.1±1.4 Hz. At this electrode, the discrimination performance of OLF-TF1 amplitude was significant (AUC = 0.89±0.07, p<0.0001) (Table 1, Figure 3). Using the cutoff value associated with the greatest Youden index, the measure of OLF-TF1 amplitude had a sensitivity of 81.8% and a specificity of 90.9% for discriminating between presence vs. absence of an EEG response to olfactory stimulation. The discrimination performance of OLF-TF1 amplitude was significantly greater than the discrimination performance of the measure of OLF-N1 (difference in AUC: 0.39±0.148, p = 0.009) and OLF-P2 (difference in AUC: 0.29±0.14, p = 0.036) peak amplitudes performed in the time domain (Table 1).

The scalp topography of OLF-TF2 was maximal at the vertex (Figure 2). At electrode Cz, the desynchronization peaked at 1190±98 ms and 9.6±1.7 Hz. The discrimination performance of OLF-TF2 amplitude was not significant (AUC: 0.57±0.13, p = 0.589) (Table 1, Figure 3).

Here, the EEG was recorded using 64 scalp channels. Considering that the EEG responses were consistently identified at electrode Cz following trigeminal stimulation and electrode Fz following olfactory stimulation, recording from these two locations could be sufficient to characterize them, in particular, in a clinical setting (to reduce the time required for setting up the recording, as well as to reduce computation time).

### Correlation between psychophysical olfactory performance and EEG response magnitude

**Figure 6:**
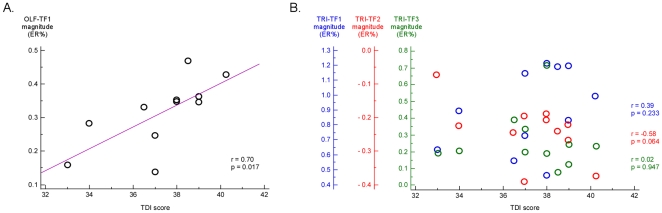
Correlation between psychophysical olfactory performance and magnitude of the significant EEG responses to chemosensory stimulation. The left panel (A) shows the scatter diagram illustrating the correlation between TDI scores and the olfactory EEG response OLF-TF1. Note the significant positive correlation between the TDI score and OLF-TF1 magnitude (r = 0.70, p = 0.017). The right panel (B) illustrates the correlation between TDI scores and trigeminal EEG responses (TRI-TF1 (blue), TRI-TF2 (red) and TRI-TF3 (green). Note the absence of significant correlation between the TDI score and these different responses to trigeminal stimulation.

Previous studies have shown a relationship between the psychophysical assessment of olfactory performance and the magnitude of chemosensory ERPs [42]. For this reason, we examined the correlation between the psychophysical TDI scores and the magnitude of the significant EEG measures identified in the time-frequency domain (TRI-TF1, TRI-TF2, TRI-TF3, OLF-TF1) (Figure 6). We found a significant correlation between TDI scores and the magnitude of the olfactory response OLF-TF1 (r = 0.70, p = 0.017). In contrast, there was no significant correlation between TDI scores and the magnitude of the different responses to trigeminal stimulation (TRI-TF1: r = 0.39, p = 0.233; TRI-TF2: r = −0.58, p = 0.064; TRI-TF3: r = 0.02, p = 0.947).

## Discussion

Using conventional across-trial averaging in the time domain, trigeminal stimulation elicited consistent ERPs, consisting of a negative wave (TRI-N1) followed by a positive wave (TRI-P2), both maximal at the vertex. The magnitude of TRI-N1 was only moderately effective at discriminating between the presence vs. absence of a trigeminal response, but the magnitude of TRI-P2 had a high discrimination performance. Contrasting with the relatively high signal-to-noise ratio of the ERPs elicited by trigeminal stimulation, olfactory stimulation elicited a clearly identifiable negative (OLF-N1) and positive (OLF-P2) peak in only a few subjects, and neither the measure of OLF-N1 amplitude nor the measure of OLF-P2 amplitude were able to discriminate between the presence vs. absence of an olfactory response.

As hypothesized, across-trial averaging in the time-frequency domain markedly enhanced the signal-to-noise ratio of the elicited responses, and revealed EEG activity that could not be identified using conventional time-domain averaging. Following trigeminal stimulation, three distinct responses were identified: TRI-TF1 which circumscribed the time-frequency representation of the phase-locked trigeminal ERP, TRI-TF2 which circumscribed a stimulus-induced desynchronization of alpha-band EEG oscillations and TRI-TF3 which circumscribed a transient non phase-locked increase in EEG power centered around 10–15 Hz. The discrimination performance of each of the three responses was highly significant, with a perfect discriminatory performance for TRI-TF1. Following olfactory stimulation, the enhancement of signal-to-noise ratio was even more pronounced. Whereas no clear olfactory ERP could be identified in the time domain, time-frequency domain analysis revealed two significant non phase-locked responses: a long-lasting increase in the amplitude of low frequencies EEG oscillations (OLF-TF1), followed by a desynchronization in the alpha-band (OLF-TF2). Most importantly, the magnitude of OLF-TF1 effectively discriminated between the presence vs. absence of an olfactory response.

### EEG responses to trigeminal stimulation

In the present study, gaseous CO_2_ was used to activate trigeminal afferents selectively [43], [44]. When applied to the nasal mucosa in short pulses, gaseous CO_2_ induces a short-lasting painful stinging sensation [45], related to the activation of Aδ- and C-fibre nociceptive afferents of the ophthalmic and maxillary branches of the trigeminal nerve, ending in the the nasal mucosa [45].

In the time domain, the obtained trigeminal ERPs (TRI-N1: 391±55 ms; TRI-P2: 554±57 ms) were similar to those reported in the literature, consisting of a negative wave peaking 320–450 ms after stimulus onset, followed by a positive wave peaking 450–800 ms after stimulus onset, both waves displaying a scalp distribution maximal at the scalp vertex [2], [46].

Time-frequency domain analysis of the obtained EEG responses revealed that, in addition to the phase-locked chemosensory ERP (TRI-TF1), trigeminal stimulation also elicited a long lasting desynchronization of ongoing alpha-band EEG oscillations (TRI-TF2) and an early, transient and non phase-locked increase in EEG power peaking approximately 350 ms after stimulus onset, centered around 10–15 Hz (TRI-TF3). The scalp topography of TRI-TF 1 and TRI-TF3 were both maximal at the scalp vertex, while the scalp topography of TRI-TF2 showed maximal desynchronization over central-parietal regions.

These stimulus-induced modulations of the magnitude of ongoing EEG rhythms are thought to result from a transient decrease or increase in the synchrony of underlying neuronal populations. Ongoing EEG oscillations in the alpha-band (8–12 Hz) are usually considered to reflect “idle rhythms” or, even, to reflect a mechanism of active cortical inhibition (for a review see [40], [47]). This view is supported by the observation that sensory stimuli induce alpha-band desynchronization over the cortical areas thought to be specifically involved in processing the stimulus (for a review see [47]). Hence, the alpha-band ERD elicited by trigeminal stimulation (TRI-TF2) may be hypothesized to reflect a stimulus-induced transient activation of neuronal populations involved in processing the trigeminal input. The functional significance of the early and brief non phase-locked increase of EEG power represented by TRI-TF3 is more speculative. It is interesting to note that a similar response has also been reported following the thermal activation of skin nociceptors [37], but also following non-nociceptive tactile, auditory or visual stimulation [48], [49], [50]. The response could reflect a stimulus-induced increase of the magnitude of ongoing EEG oscillations (ERS), but could also reflect a stimulus-evoked ERP affected by a significant amount of latency jitter [25], [37]. Indeed, ERP components subject to a significant amount of temporal jitter will appear as non phase-locked relative to stimulus onset, and this dephasing will affect more greatly components of higher frequency. Importantly, the latency of TRI-TF3 was slightly but significantly more precocious than the latency of the phase-locked ERP response (TRI-TF1), thus suggesting that TRI-TF3 reflected earlier and, hence, potentially more specific aspects of trigeminal cortical processing.

### EEG responses to olfactory stimulation

In the present study, activation of olfactory afferents was achieved using 2-phenylethanol, which is usually described as a pleasant floral odorant.

After time-domain averaging, olfactory ERPs were identifiable in only a few subjects. When present, the response consisted of a negative wave (OLF-N1: 397±27.48 ms) followed by a positive wave (OLF-P2: 616±109.28 ms), maximal at the vertex, compatible with what has been described previously [2], [30], [31]. Crucially, the fact that we could not identify olfactory ERPs in a significant number of normosmic subjects was entirely expected, as previous studies have also reported that the signal-to-noise ratio of olfactory ERPs is relatively poor [10], [26]. For example, Lötsch and Hummel [10] stated that they were unable to identify any reproducible olfactory ERP in approximately 30% of normosmic subjects. This was confirmed by our finding that neither OLF-N1 amplitude nor OLF-P2 amplitude discriminated significantly between presence vs. absence of an olfactory response.

Compared to trigeminal ERPs, several factors could contribute to the lower signal-to-noise ratio of olfactory ERPs. First, possibly because of its nociceptive nature, trigeminal stimulation is usually perceived as much more sharp and intrusive than olfactory stimulation. Hence, because chemosensory ERPs are thought to at least partly reflect cortical processes involved in arousal and/or stimulus-triggered attentional reorientation [51], the relatively low amplitude of olfactory ERPs could be due to their relatively low salience. In addition, it should be noted that stimuli were delivered using a constant 30-s inter-stimulus interval (ISI), such as in several other studies [52], [53], [54], [55]. The regular occurrence of the stimulus could thus have induced some amount of response habituation [26]. Future studies could examine whether the use of variable ISIs could further increase the signal-to-noise ratio of the elicited responses. Second, residual odorant molecules could contaminate the experimental environment, leading to an important reduction of the signal-to-noise ratio of the actual stimulus. Third, the cellular mechanisms involved in olfactory signal transduction could be less phasic and stationary than those underlying the transduction of trigeminal input and, hence, may be less suited for the recording of time-locked EEG responses.

Nevertheless, time-frequency analysis of the EEG signals following olfactory stimulation did reveal a number of significant, non phase-locked EEG responses, consisting in a long-lasting increase in the amplitude of EEG frequencies centered around 5 Hz (OLF-TF1), followed by a desynchronization of alpha-band oscillations (OLF-TF2). The scalp topography of OLF-TF1 was maximal at the vertex, and similar to the scalp topography of the olfactory ERP. Hence, both responses could reflect the same stimulus-evoked cortical activity, which would be markedly dephased by a large amount of temporal jitter, thus explaining why the response appears largely non phase-locked relative to stimulus onset, and why its average duration appears long and ill-defined.

### Discrimination performance

As expected from their relatively high signal-to-noise ratio, the TRI-N1 and TRI-P2 waves of trigeminal ERPs, characterized using conventional time-domain averaging, were able to discriminate between the presence vs. absence of a trigeminal response with a high sensitivity and specificity (TRI-N1: p = 0.038, Sensitivity: 81.2%, Specificity: 63.6%, TRI-P2: p<0.0001, Sensitivity: 81.2%, Specificity: 81.2%). When characterizing these responses in the time-frequency domain, discrimination performance of these responses was even greater: in our sample of healthy subjects, the magnitude of TRI-TF1 was able to discriminate between presence vs. absence of a response with a sensitivity and specificity of 100%. However, this improvement of discrimination performance was not significant, mainly because trigeminal ERPs can already be very effectively identified in the time domain.

The discrimination performance of olfactory ERPs identified using conventional time-domain averaging was very poor and, within the present study conditions, close to chance level (OLF-N1: p = 0.975, Sensitivity: 36.4%, Specificity: 31.8%, OLF-P2: p = 0.429, Sensitivity: 45.4%, Specificity: 90.9%). This observation confirms the results of previous studies [10]. In contrast, the discrimination performance of the EEG responses to olfactory stimulation identified in the time-frequency domain was markedly and significantly higher. Indeed, in our sample of normosmic subjects, the low frequency non phase-locked EEG response characterizing OLF-TF1 was able to discriminate between presence vs. absence of a response with a sensitivity of 81.8% and a specificity of 90.9% (p<0.0001).

### Relationship with perception and clinical usefulness

**Figure 7:**
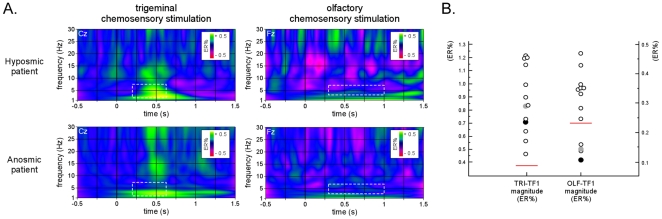
Clinical usefulness of time-frequency analysis of chemosensory ERPs. A. Time-frequency representation of the non-phase locked EEG responses to trigeminal and olfactory chemosensory stimulation (CWT-SINGLE; see Methods) in one hyposmic patient (TDI = 23) and one anosmic patient (TDI = 14). Signal amplitude is expressed as percentage increase or decrease relative to baseline (−0.4 to −0.1 s) (trigeminal stimulation: electrode Cz; olfactory stimulation: electrode Fz). B. Magnitude of TRI-TF1 and OLF-TF1 measured in the hyposmic patient (grey dot), the anosmic patient (white dot) and the 11 healthy controls (black dot). The horizontal red line indicates the cutoff amplitude value associated with the greatest Youden index. Note that the magnitude of OLF-TF1 measured in the hyposmic and anosmic patients are both below the cutoff value. In contrast, note that the magnitude of TRI-TF1 measured in the two patients was similar to those measured in the healthy controls.

The finding that the psychophysical assessment of olfactory performance (TDI scores) correlated significantly with the magnitude of the EEG response to olfactory stimulation (OLF-TF1) but not with the different EEG responses to trigeminal stimulation further supports the view that this response relates to olfactory perception, and highlights its potential clinical usefulness (see also Figure 7).

Future studies including larger cohorts of patients with olfactory disorders and age-matched healthy controls are needed to confirm the clinical usefulness of our approach. Of particular interest would be to examine the relationship between functional measures of olfaction (psychophysical and electrophysiological assessment of olfaction) and structural measures of the olfactory system (e.g. MRI-based evaluation of olfactory bulb volume and/or olfactory sulcus depth) [56].

In summary, we show that, within the present study conditions, time-frequency analysis of the EEG activity triggered by trigeminal and olfactory stimulation can disclose responses that cannot be identified using conventional time-domain averaging. Because the non-phase locked responses identified using time-frequency analysis are likely to reflect different aspects of the cortical processing of chemosensory stimuli, the approach provides a more complete view of how odors are represented in the human brain.

Furthermore, we show that our approach markedly enhances the signal-to-noise ratio of the elicited EEG responses, in particular, the responses elicited by olfactory stimulation. The ability to detect, with a high sensitivity and specificity, the EEG responses to olfactory stimulation is not only of interest for neuroscientists aiming to understand the cortical processes involved in the perception of odors in humans. Indeed, our approach could also constitute the basis for a clinical diagnostic tool for the clinical evaluation of patients complaining of olfactory disorder, but also for the early differential diagnosis of certain neurodegenerative diseases thought to be associated with olfactory dysfunction. However, future studies will be needed to assess fully the clinical usefulness of this approach.

## Methods

### Participants

After obtaining written informed consent, experiments were performed in 11 healthy normosmic volunteers (3 females and 8 males), aged between 24 and 30 years. Investigations were approved by the local Ethics Committee (Comité d'Ethique Biomédicale Hospitalo-Facultaire, Université catholique de Louvain, Faculty of Medecine, N°: B40320108777). In order to ensure that participants were normosmic, orthonasal olfactory function was assessed using the validated Sniffin' Sticks test [57], [58]. In this test, odors are presented to the subjects using felt-tip pens placed approximately 2 cm in front of both nostrils, as follows. First, the “olfactory threshold” (T) is assessed using n-butanol presented by means of a single staircase, using stepwise dilutions in a row of 16 felt tip pens. Second, “odor discrimination” (D) is assessed by asking subjects to perform a triple forced choice using 16 pairs of odorant. Third, “odor identification” (I) is assessed by asking subjects to identify 16 individual odors by performing a forced choice from a list of four verbal descriptors. Olfactory threshold (T), discrimination (D) and identification (I) scores were then added together to give the TDI score [57], [58]. All subjects were considered as normosmic according to the Sniffin' Sticks test (TDI scores ranging from 33 to 40.25; 37.2±2.16).

### Stimuli

Chemosensory stimuli were produced by an air-dilution olfactometer (OM2S, Burghart Medical Technology, Wedel, Germany). The device is able to deliver brief pulses of odorant embedded within a constant airflow. The rapid switching between the odor and the control airflow is based on a vacuum line. During stimulation, the airflow (8 l/min), temperature (36°C) and humidity (80% relative humidity) remain strictly unchanged, thus avoiding any concomitant stimulation of mechanical or heat sensitive trigeminal receptors. Trigeminal stimuli were generated by gaseous CO_2_ (55% v/v) used to activate trigeminal afferents, and olfactory stimuli were generated by 2-Phenylethanol (50% v/v) used to activate olfactory afferents [5], [59]. The stimuli were delivered through a Teflon™ tube placed in the right nostril, just behind the nasal valve, pointing towards the olfactory cleft. Stimulus duration was 200 ms, with a rising time of 20 ms.

### Procedure

Before the experiment, subjects were familiarized with the experimental surrounding, the material used for the psychophysical assessment of olfaction, as well as the olfactory and trigeminal stimuli used to elicit the chemosensory ERPs. During the experiment, olfactory and trigeminal stimuli were presented in alternation. Each type of stimulus was repeated 60 times. Inter-stimulus time interval between each stimulus was 15 s. Hence, the inter-stimulus interval between two stimuli of the same type was 30 s. Subjects were instructed to breathe through the mouth and to perform velo-pharyngeal closure to avoid any respiratory airflow in the nasal cavity during stimulus presentation [60]. Subjects were also instructed to keep their eyes open during the recording.

### Electroencephalographic recording

The electroencephalogram was recorded continuously from 64 Ag/AgCl electrodes placed on the scalp according to the International 10/10 system (Waveguard64 cap, Cephalon A/S, Denmark). Scalp signals were recorded using an average reference. Ocular movements and eye-blinks were recorded using two additional bipolar surface electrodes placed at the upper-left and lower-right sides of the left eye. Impedance was kept below 5 kOhm. Signals were amplified and digitized at a 1000 Hz sampling rate (64-channel ASA-LAB EEG system, Advanced Neuro Technologies, The Netherlands).

### Data preprocessing

All EEG processing steps were carried out using BV Analyzer 1.05 (Brain Products, Germany), Letswave (http://nocions.webnode.com/letswave) [25] and EEGLAB (http://sccn.ucsd.edu/eeglab).

After referencing to the left (M1) and right (M2) mastoids, and after band-pass filtering using a 0.3 to 30 Hz Butterworth zero phase filter, the continuous EEG recordings were segmented into 2.0 s long EEG epochs ranging from −0.5 to +1.5 s relative to stimulus onset.

After baseline correction (reference interval: −0.5 to 0 s), an Independent Component Analysis (ICA) was performed to remove electro-ocular artifacts, using the runica algorithm as implemented in EEGLAB [61], [62]. Artifact-free epochs were generated by removing the ICs capturing clear electro-ocular artifacts (time course typical of eye blinks, frontal scalp topography) [63], [64]. Finally, epochs with amplitude values exceeding±100 µV (i.e. epochs likely to be contaminated by an artefact) were rejected (22±11% of the total number of epochs).

### Across-trial averaging in the time domain

For each subject, separate average waveforms were computed for trigeminal and olfactory stimulation. Within these average waveforms, two distinct peaks were measured at electrode Cz, as described in previous studies [5], [11], [59], [65], [66]. For trigeminal chemosensory ERPs, N1 was defined as the most negative peak between 320 and 450 ms (TRI-N1) and P2 was defined as the most positive peak between 450 and 800 ms (TRI-P2). For olfactory ERPs, N1 was defined as the most negative peak between 320 and 450 ms (OLF-N1) and P2 was defined as the most positive peak between 450 and 800 ms (OLF-P2). Peak latencies were expressed relative to stimulus onset. Peak amplitudes were expressed relative to baseline.

### Across-trial averaging in the time-frequency domain

A time-frequency (TF) representation based on the continuous Morlet wavelet transform (CWT) of EEG epochs was used to characterize the amplitude of oscillatory activity as a function of time and frequency. The Morlet wavelet consists in a complex exponential function localized in time by a Gaussian envelope. The initial spread of the Gaussian wavelet was set to 2.5/πω0 (ω0 being the central frequency of the wavelet; see also [25], [37]). Explored frequencies ranged from 0.3 to 30 Hz in steps of 0.3 Hz.

To obtain a time-frequency representation of trigeminal and olfactory ERPs, the time-frequency transform was first applied to the single-subject ERP waveforms obtained after time-domain averaging (CWT-AVERAGE). Because time-domain averaging cancels out all signal changes that are not strictly stationary across trials, this transform revealed only stimulus-induced EEG changes that were phase-locked to the stimulus onset (i.e. ERPs).

To obtain a time-frequency representation of both phase-locked and non phase-locked EEG responses to trigeminal and olfactory stimulation, the time-frequency transform was then applied to each single EEG epoch (CWT-SINGLE). For each subject and stimulus type, single-trial TF maps expressing signal amplitude were then averaged across trials. Because this approach yields a time-frequency map of the average oscillation amplitude regardless of phase, it enhanced both phase-locked (i.e. ERPs) and non phase-locked (i.e. ERD, ERS and ERPs affected by a significant amount of latency jitter) stimulus-induced changes in EEG oscillation amplitude.

For each estimated frequency, CWT-AVERAGE and CWT-SINGLE time-frequency maps were expressed relative to baseline (pre-stimulus interval ranging from −0.4 to −0.1 s relative to stimulus onset), as follows: *ER%_t,f_ = (A_t,f_−R_f_)/R_f_*, where *A_t,f_* is the signal amplitude at a given latency *t* and frequency *f*, and *R_f_* is the signal amplitude at the frequency *f*, averaged within the pre-stimulus reference interval.

To assess the significance of the relative increases and decreases of signal amplitude observed in the group-level average time-frequency maps, for each time-frequency bin, a one-sample t-test against zero was performed using the amplitudes estimated in each subject. This yielded, for each type of stimulus, a time-frequency map highlighting the regions where the EEG signal deviated significantly from baseline (p<0.05). These statistical maps were then used to define a number of regions of interest (ROI), circumscribing stimulus-induced EEG responses. For each subject, maximum or minimum amplitude values within each ROI were used as summary values estimating response magnitude.

### Discrimination performance

The aim of the present study was to characterize the phase-locked and non phase-locked EEG responses to olfactory and trigeminal stimulation and, most importantly, to evaluate the reproducibility and robustness of these responses when recorded at individual level, in order to assess their clinical usefulness.

For this purpose, an additional dataset was created by segmenting the original continuous EEG recordings from −2.5 to −0.5 s relative to stimulus onset. Whereas the original dataset (STIM) was expected to contain EEG responses triggered by the olfactory or trigeminal stimulus, this additional dataset (NOSTIM) was expected to contain only background EEG activity. Exactly the same analysis was applied to this NOSTIM dataset. For each stimulus type, Receiver Operating Characteristic (ROC) curves were then constructed to examine and compare the ability of each different measures of the magnitude of the EEG responses to chemosensory stimulation to discriminate between STIM and NOSTIM epochs, i.e. to discriminate between the stimulus-evoked EEG responses and background noise. These analyses were performed using MedCalc v. 11.5. (MedCalc Software, Belgium). The area under the ROC curve (AUC) was used as an index of discriminatory performance. An AUC of 0.5 would indicate random performance, whereas an AUC of 0 and 1 would denote perfect performance. For each measure, to assess the ability to distinguish between presence vs. absence of a response, it was thus examined whether the AUC was significantly different from 0.5 [67], [68]. When significant (p<0.05, uncorrected for multiple comparisons), the cut-off value (J) associated with the greatest Youden index (Y = sensitivity+specificity-1) was chosen as decision criterion [69], [70]. This cutoff value corresponds to the point on the ROC curve that is farthest from the diagonal line. Finally, to compare the discrimination performance of the different EEG measures, obtained ROC curves were compared using the nonparametric approach for comparing the areas under two or more correlated ROC curves described by Delong et al. [71].

To examine the involvement of the different EEG responses to olfactory and trigeminal stimulation in chemosensory perception, the relationship between their magnitude and the psychophysical TDI scores assessing olfactory performance was assessed using the Pearson's correlation coefficient.
